# Mental Well-Being: 2010–2018 Trends among Italian Adolescents

**DOI:** 10.3390/ijerph19020863

**Published:** 2022-01-13

**Authors:** Michela Bersia, Paola Berchialla, Lorena Charrier, Patrizia Lemma, Alberto Borraccino, Paola Nardone, Daniela Pierannunzio, Silvia Ciardullo, Rosanna Irene Comoretto, Paola Dalmasso

**Affiliations:** 1 Department of Public Health and Pediatrics, University of Torino, Via Santena 5 bis, 10126 Torino, Italy; michela.bersia@edu.unito.it (M.B.); patrizia.lemma@unito.it (P.L.); alberto.borraccino@unito.it (A.B.); rosannairene.comoretto@unito.it (R.I.C.); paola.dalmasso@unito.it (P.D.); 2 Post Graduate School of Medical Statistics, University of Torino, Via Santena 5 bis, 10126 Torino, Italy; 3 Department of Clinical and Biological Sciences, University of Torino, Regione Gonzole 43, 10043 Orbassano, Italy; paola.berchialla@unito.it; 4 National Centre for Disease Prevention and Health Promotion, Italian National Institute of Health, Viale Regina Elena 299, 00161 Rome, Italy; paola.nardone@iss.it (P.N.); daniela.pierannunzio@iss.it (D.P.); silvia.ciardullo@iss.it (S.C.)

**Keywords:** mental health, adolescents, well-being, temporal trend, HBSC, Dual Factor Model, life satisfaction, psychological health complaints

## Abstract

(1) Aims: To explore temporal trends 2010–2018 of well-being among Italian adolescents and to evaluate potential explanatory factors. (2) Methods: Italian nationality representative samples of students aged 11, 13, and 15 years were recruited in 2010, 2014, and 2018; Health Behaviour in School-aged Children (HBSC), for an overall number of 165,000 teenagers. Multivariable logistic regression models were performed to fit the trends over time of life satisfaction (LS), psychological (PSY-HC) and somatic health complaints (SOM-HC) considering the contextual factors: school work pressure, social support (family, school, peers), socioeconomic status, geographic area, and immigration background; (3) Results: From 2010 to 2018 while LS was steady, health complaints increased, mainly for PSY-HC, in all age and gender groups. Trend of PSY-HC affected mainly 15-years-olds: rates among boys varied from 29.6% to 35.9% (OR: 1.13, 95%CI: 1.02–1.25); among girls from 49.1% to 63.3% (OR: 1.56, 95%CI: 1.42–1.72). High school work pressure and poor social support play a central role in worsening well-being outcomes; (4) Conclusions: Our findings pictured a remarkable worsening trend of teenagers’ well-being, especially among 15-year-old girls. Further research will be required to investigate this breaking up of the connection between psychophysical symptomatology and cognitive perception of life satisfaction.

## 1. Introduction

### 1.1. The Concept of Health as Well-Being

In the 20th century, the concept of health evolved to include the notion of well-being: according to the World Health Organization (WHO), health is a state of complete physical, psychological, and social well-being, and not just the absence of disease and infirmity [1]. Therefore, there cannot be health without mental health: medically ill people with an optimistic attitude have better outcomes (e.g., lower rates of death from cardiovascular disease) than those with high levels of pessimism [2].

Moreover, in the last 20 years, mental health has become central for its impact on public health: 1996 WHO projections indicated that by 2020 depression would be the second leading cause of illness, emphasizing adolescence as a fundamental period in forming the features of adult mental health [3,4,5,6,7,8].

### 1.2. Mental Health during Adolescence: Theorization and Consequences

The Bronfenbrenner Bioecological Model of Human Development underlines how puberty could be a crucial period for adolescent mental health [3,4]. It accounts for different system levels—individual, microsystem, mesosystem, and macrosystem—and considers the maturational stage of the adolescent into his/her social background.

To the best of our knowledge, it was confirmed that measures of well-being are worse in older adolescents than in younger ones [9]. Similarly, boys show more favorable measures of happiness, confidence, helplessness, and lower psychological complaints than girls [9,10,11,12].

Thus, from a very young age, well-being can be considered a delicate balance of different determinants: if some of them wobble, the entire building can collapse, with serious consequences in term of both internalizing problems (i.e., depression, anxiety, sleep disorders, or withdrawal symptoms) and externalizing behaviors (i.e., aggression, oppositional disorders, or delinquency) [13].

### 1.3. Trends and Determinants

WHO estimates that 10–20% of adolescents globally experience mental illness, largely underdiagnosed and undertreated [14]. In support of this estimation, the 2018 Health Behaviour in School-aged Children survey (HBSC) observed a prevalence of multiple psychosomatic health complaints among European adolescents (30–41%) and a mean life satisfaction of 7.4–8.3 on a 0–10 scale [15].

However, the identification of trends in adolescents’ well-being is controversial: a cross-national study revealed a not homogeneous picture among European countries [16], as well as a recent worldwide meta-analysis showed a mild increase between the 1980s and 2000s and an overall stability up to the 2010s [17].

Nevertheless, an increasing trend in psychological health complaints has already been observed in the last 20 years, especially among older girls, in Canada [18] and in several European countries, like Sweden [19], Norway [10,20], and the UK [21], while life satisfaction showed an unclear evolution [22,23].

Mechanisms involved in this increasing trend have not been entirely understood yet.

A previous Sweden study found that the increasing trend in psychological complaints can be mainly associated with schoolwork pressure [24]. Recently, the importance of family and school has been emphasized in different studies on the role of scholar stress and low social support (i.e., poor family, peer, teacher relationships) towards well-being impairment [24,25,26,27,28].

In particular, Levin found an important positive association between mother–child communication and young people’s life satisfaction, especially among girls [26]. Also for immigrant adolescents, a positive scholar environment is a protective factor for better mental health outcomes [29]. Similarly, in the majority of the countries, lower socioeconomic status (SES) or worries for family finances were associated with worse mental outcomes, in terms of both life satisfaction and psychological complaints [18,28,30,31].

As these mechanisms are profoundly rooted in the socio-cultural context of each country, it is difficult to generalize the results already observed. In Italy, a slight decline in mental well-being has been observed in the last decades [16]. However, country-specific studies are needed to better understand circumstances, processes, and mechanisms involved in this complex public health issue.

The aim of this study was to explore temporal trends of health complaints and well-being among Italian adolescents from 2010 to 2018 and to evaluate potential explanatory factors.

## 2. Materials and Methods

### 2.1. Study Population

The present study was based on data from the Italian Health Behaviour in School-Aged Children (HBSC) carried out in 2010, 2014, and 2018. The HBSC is an international multicenter survey that runs every four years using a standardized research protocol to investigate health-related behaviours in adolescents (Health Behaviour in School-aged Children; World Health Organization collaborative cross-national survey; available from www.hbsc.org accessed on 14 December 2021).

For each of the three survey rounds, nationally representative samples of 11-, 13-, and 15- years old were recruited from school classes throughout Italian regions. The school class was the primary sampling unit, drawn by a stratified systematic cluster sampling from a list of all public and private schools obtained from the Ministry of Education. The Italian HBSC study included around 2500 classes in 2010, 3300 in 2014, and 4100 in 2018: the response rates were, respectively, 96% in 2010 [32], 90% in 2014 [33], and 86% in 2018 [34] of all sampled classes. Data were collected in classroom settings through standardized, self-filled questionnaires on social background, health indicators, and health-related behaviours. Anonymity and confidentiality of all participants were ensured. Participation was voluntary, and parental opt-out consent was obtained.

Italian HBSC study protocols and questionnaires were formally approved by Ethics Committees of the University of Turin (2014) and of the Italian National Institute of Health (2010 and 2018). A detailed description of the aims, theoretical framework, and protocol of the international and Italian HBSC study can be found elsewhere [32,33,34].

### 2.2. Measures

Adolescent mental well-being was measured by two instruments:

- Life Satisfaction (LS) was assessed with the Cantril ladder [35], a reliable instrument for subjective well-being among adolescents [36]. Participants were asked to rate their life satisfaction using a visual analogue scale (range 0–10): higher the score, greater the feeling of life satisfaction. Respondents were asked to indicate the ladder step at which they would place their lives at present. The findings presented here were categorized as low (0–8)/high LS (≥9) [37].
- Psychosomatic Health Complaints (HC) were evaluated through the HBSC Symptom Checklist (HBSC-SCL), a non-clinical measure consisting of eight items (headache, stomachache, backache, feeling low, irritability or bad-tempered, feeling nervous, sleeping difficulties, and dizziness). Adolescents indicated how often they had experienced each complaint over the last six months. Response options for each symptom ranged from about every day to rarely or never. According to the binary cut-off used in the international report [15], findings presented here were categorized as participants with multiple (two or more) health complaints more than once a week/other (reference category). This instrument has adequate test-retest reliability and psychometric properties [38,39] and is commonly used to assess adolescent mental well-being [40]. HBSC-SCL can be thought of as constituting two dimensions: somatic (SOM-HC) (i.e., headache, stomachache, backache, and dizziness) and psychological complaints (PSY-HC) (i.e., feeling low, irritability, feeling nervous, and sleeping difficulties) [41]. SOM-HC and PSY-HC were dichotomized as participants perceiving multiple (two or more) complaints more than once a week/other (reference category).

### 2.3. Explanatory Variables

- Gender and age. Participants were asked to indicate whether they were a boy or a girl, as well as their month and year of birth.
- Geographic area. The geographic area is derived from the school address and classified into Northern, Central, and Southern Italy [42].
- Socioeconomic Status (SES) was measured according to the family affluence scale (FAS), which has been recognized as a reliable indicator of family wealth [43]. The scale consisted of four items: family car ownership, whether adolescents have their own bedroom, number of holidays trips taken in the last year, and number of computers owned by the family. The obtained score (0–7) was categorized on a 3-point ordinal scale: low (0–3), medium (4–5), and high (≥6) SES.
- Schoolwork Pressure. Adolescents responded to the question, “How pressured do you feel by the schoolwork you have to do?”. The response options available were “not at all” (1), “a little” (2), “some’’ (3), and “a lot” (4). The responses were re-coded into high (3–4) and low (1–2) pressure, as classified by the international HBSC report [15].
- Classmate Support was measured by three items: (1) “The students in my class enjoy being together”, (2) “Most of the students in my class are kind and helpful”, and (3) “Other students accept me as I am”. Response categories for all the above items ranged from strongly agree (1) to strongly disagree (5). Original codes were reversed: strongly disagree (0) to strongly agree (4), and a sum-score was generated for each domain (range 0–12) and then divided by three. The resulting average score was categorized as low (<2.5) or high (≥2.5) classmate support [44].
- Family Support was assessed by two items. Young people were asked how easy it is for them to talk to their mother or father about things that really bother them. Response options ranged from very easy to very difficult. In this study, we dichotomized finding as easy or very easy/difficult or very difficult. Communication in the family is an indicator of social support and of the family’s connectedness [45,46,47].
- Immigration background was defined by the country of birth of the adolescents and their parents. If at least one parent was born abroad, adolescents were classified as having an immigrant status [45].
- Family structure was determined by asking adolescents to identify the people who live, most of the time, in the same house with them. Adolescents were then classified as “living with both biological parents” (traditional family) or “living with other adults” (non-traditional family) [48].

### 2.4. Statistical Analyses

Descriptive characteristics of the sample were reported by survey year. Differences were tested with the corrected weighted Pearson Chi square test.

**Figure A1:**
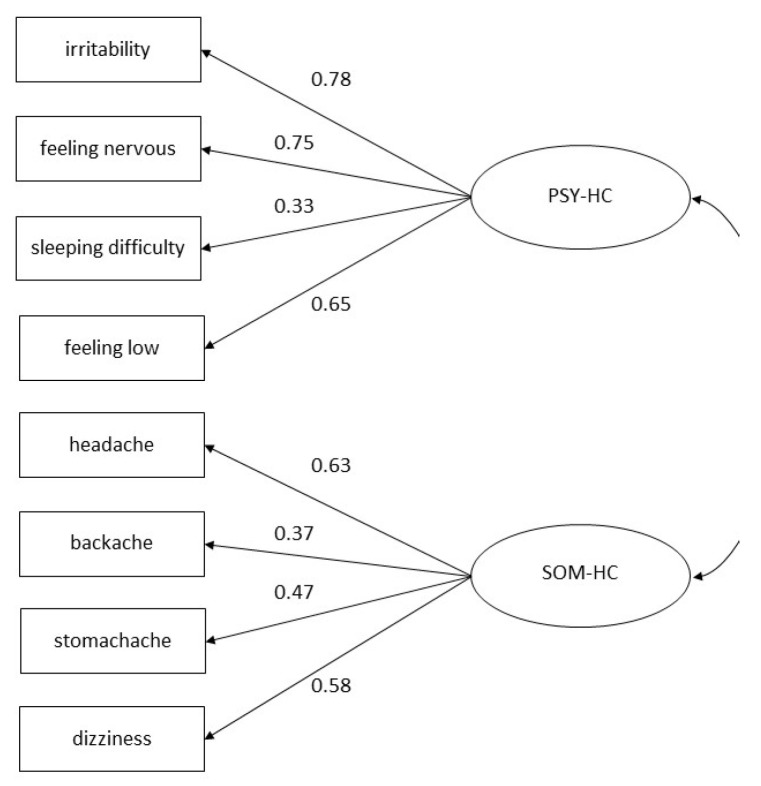
Confirmatory Factor Analysis on health complaints. Results from the double factor (PSY-HC and SOM-HC) model for the dichotomized variable. Standardized coefficients of correlation between latent and observed variables. Abbreviations. HC: ≥2 health complaints more than once a week; PSY-HC: ≥2 psychological health complaints more than once a week; SOM-HC: ≥2 somatic health complaints more than once a week.

Confirmatory factor analysis (CFA) was conducted. For health complaints as a continuous variable, Structural Equation Modelling (SEM) was performed on a single factor model (i.e., all items of the HBSC-SCL were supposed to be represented by the same factor) in comparison with a model comprising two correlated factors (psychological and somatic). Similarly, Generalized Structural Equation Modelling (GSEM) was used for a single factor and a two-factor model considering health complaints as a dichotomized variable. According to AIC (Akaike Information Criterion), and BIC (Bayesian Information Criterion) measures of fit (see Table A1 in Appendix A), the two-factor model for the dichotomized variable fitted the data best, so it has been adopted as an outcome variable in trend estimation [11,49]. Figure A1 shows results from the two-factor model for the dichotomized variable. Psychological and somatic symptoms, the two factors of the model, are two correlated dimensions [49].

The trends of the prevalence of mental well-being from 2010 to 2018 were estimated using several multivariable logistic regression models considering LS and HC (as overall and PSY-HC and SOM-HC (yes/no)) as the dependent variables and the survey year, Family Affluence Scale, geographic area, schoolwork pressure, classmate support, mother support, father support, family structure, and immigration background as independent variables. In the regression models, survey year was entered first as a categorical variable to estimate differences between rounds; second, as a continuous variable to estimate an overall trend per year in the considered time period.

An interaction term between gender and the survey year was also included in the model to examine whether the trends were moderated by gender. Because of the interaction, the analyses were stratified by gender and age class.

In the analyses, the effect of the survey design, including stratification, clustering, and weighting were considered. A statistical significance level of 5% was set up. Analyses were carried out using STATA v16.1 (StataCorp LLC: College Station, TX, USA).

## 3. Results

More than 165,000 students participated in the survey in the three waves (35.6% in 2010, 28.8% in 2014, and 35.6% in 2018) with similar gender and age distribution among the three time-points. Table 1 gives an overview of the characteristics of the total sample by survey wave. Across the surveys, the percentage of youths with missing responses ranged from 0% (gender, age, geographic area) to 5.2% (easy communication with father).

Low SES index (measured according to the FAS) increased from 13.3% in 2010 to 22.3% in 2018 as well as non-traditional family structure (from 14.7% to 18.0%). Despite an important increase in schoolwork pressure (41.8–54.9% in the period 2010–2018) being observed, social support remained substantially stable (family support) or even decreased (classmate support, from 81.0% to 75.7%).

**Figure 1:**
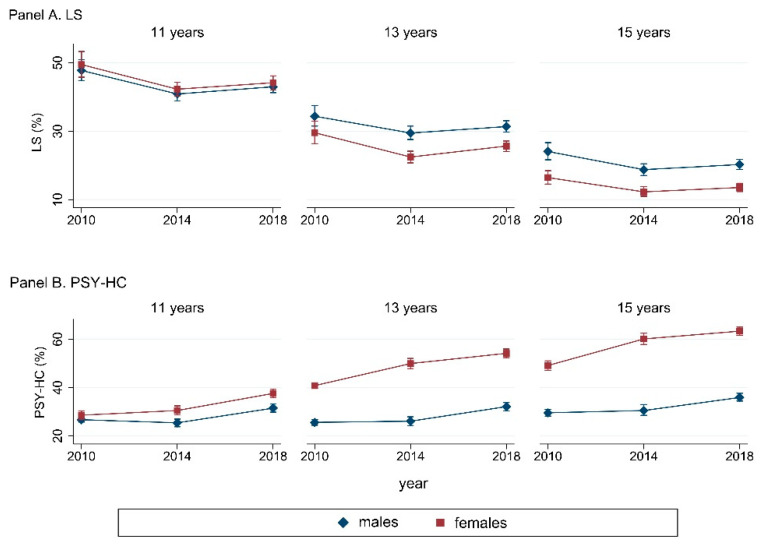
Prevalence (% (95%CI)) of LS (**Panel A**) and PSY-HC (**Panel B**), stratified by gender and age groups. Abbreviations: LS: life satisfaction ≥9; PSY-HC: ≥2 psychological health complaints more than once a week.

Adolescents perceived more psychosomatic health complaints (HC) in 2018 (52.2%) compared to 2010 (47.3%), and it was seen mainly for psychological symptoms (PSY-HC). Considering psychological and somatic symptoms (SOM-HC) as separated, mental illness complaints seemed to be the major issue. As shown in Figure 1, an increasing gender gap was observed over time: PSY-HC grew more among 13- (55.9–64.3%) and 15-year-old females (63.5–72.9%) than among boys of the same age. However, it was not true for life satisfaction (LS), which showed an overall stability over time. For a more detailed description of endpoint variables, see Table A2, included in the Appendix A.

### 3.1. Trend in Well-being Measures (LS, HC, PSY-HC and SOM-HC): Gender and Age Disparities

Results from the multivariable regression analysis were reported in Table 2.

From 2010, the increasing trend per year in psychosomatic HC was statistically significant among older adolescents, weaker for 13-year-old boys (yearly OR: 1.02; 95%CI: 1.00–1.03) and girls (OR: 1.03; 95%CI: 1.02–1.04), stronger for 15-year-old females (OR: 1.04; 95%CI: 1.02–1.05) and boys (OR: 1.02; 95%CI: 1.01–1.04).

**Figure 2:**
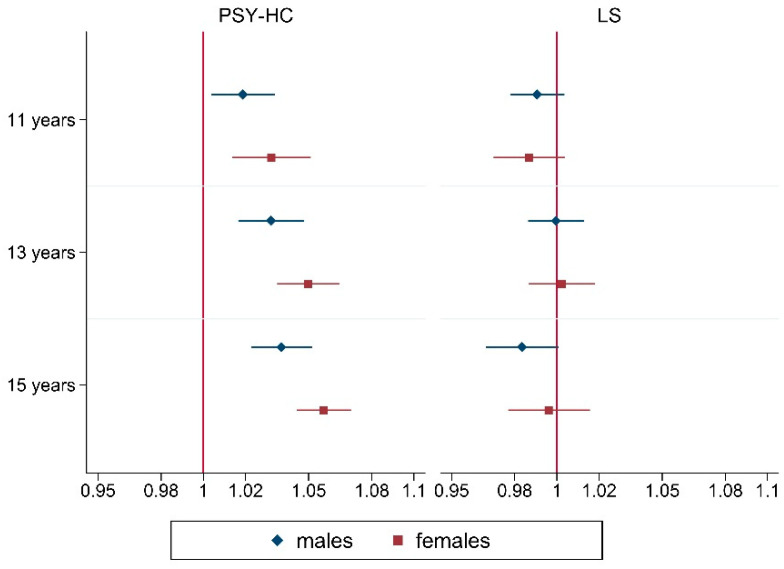
Yearly adjusted trends of PSY-HC and LS from 2010 to 2018, stratified by gender and age groups. Results from multivariable logistic regression (OR (95%CI)). Abbreviations. LS: life satisfaction ≥9; PSY-HC: ≥2 psychological health complaints more than once a week. Adjustment variables: survey year, family affluence scale, schoolwork pressure, classmate support, mother support, father support, family structure, immigration background, geographic area.

The increasing prevalence of HCs referred mainly to PSY-HC, in all age and gender groups: as represented in Figure 2, respectively, in 11-, 13-, and 15-year olds a mean significant growth of 2% (OR: 1.02; 95%CI: 1.00–1.03), 3% (OR: 1.03; 95%CI: 1.02–1.05) and 4% (OR: 1.04; 95%CI: 1.02–1.05) per year was seen from 2010 to 2018 among boys, while a mean raise of 3% (OR: 1.03; 95%CI: 1.01–1.05), 5% (OR: 1.05; 95%CI: 1.04–1.07) and 6% (OR: 1.06; 95%CI: 1.05–1.07) was observed among girls. In other words, among 11–15 years old girls, respectively, a 26% (OR: 1.26; 95%CI: 1.11–1.44)–56% (OR: 1.56; 95%CI: 1.42–1.72) mean raise of PSY-HC was registered in 2018, compared to 2010, more than among boys, with a mean growth of 13% (OR: 1.13; 95%CI: 1.02–1.25)–33% (OR: 1.33; 95%CI: 1.20–1.48).

As shown in Table A3 (Appendix A), among psychological symptoms, sleeping difficulty increased the most, in mean between 3% (OR: 1.03; 95%CI: 1.00–1.06) and 9% (OR: 1.09; 95%CI: 1.07–1.10) per year from 2010 to 2018 across gender and age groups. Also, irritability and feeling nervous showed a relevant raise, respectively, between 3% (OR: 1.03; 95%CI: 1.01–1.04)–7% (OR: 1.07; 95%CI: 1.05–1.08) and 2% (OR: 1.02; 95%CI: 1.00–1.04) and 5% (OR: 1.05; 95%CI: 1.04–1.07) per year, feeling low did not present a statistically significant trend in the last decade.

In contrast to PSY-HC, we found a slight worsening in SOM-HC, which achieved statistical significance among older females.

LS did not show a statistically significant trend from 2010 to 2018, except for a slight decrease from 2010 to 2014, in both gender and age groups.

All the measures of well-being showed an increasing gender gap: girls were always more affected than boys and differences by age already grew from 11 years old for HC, PSY-HC and SOM-HC, while it happened later for LS (from 13 years old).

### 3.2. Social Determinants of Well-Being

**Figure 3:**
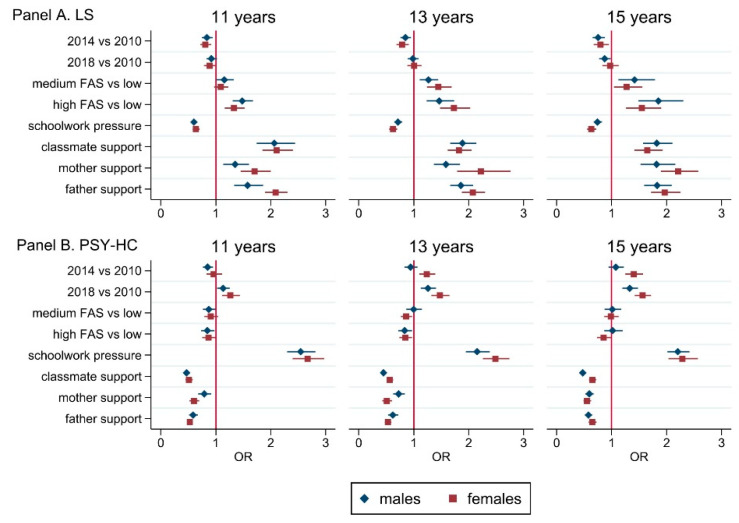
Multivariable regression analysis (OR (95%CI)) performed on LS (**Panel A**) and PSY-HC (**Panel B**), stratified by gender and age groups. Abbreviations. LS: life satisfaction ≥ 9; PSY-HC: ≥2 psychological health complaints more than once a week. Adjustment variables: survey year, family affluence scale, schoolwork pressure, classmate support, mother support, father support, family structure, immigration background, geographic area.

Figure 3 shows the association between social determinants (individual and family variables) and well-being outcomes (see also Table A4 in the Appendix A).

Schoolwork pressure, family structure, easy communication with mother and father, and classmate support were all predictors of well-being. Among variables included in the multivariable regression models, schoolwork pressure showed the strongest association with health complaints, especially psychological. Family easy communication and peer support have been significantly associated with better well-being outcomes, mostly for psychological symptoms and among females. Higher socioeconomic status was seen to affect LS strongly, especially for 15-year-old boys (OR: 1.85; 95%CI: 1.49–2.30) and girls (OR: 1.55; 95%CI: 1.26–1.90), in comparison to lower SES. Similarly, HCs were registered significantly lower among adolescents with high SES families, psychological ones (feeling low, irritability, feeling nervous) in all age and gender groups, and somatic ones among 15-year olds, both boys (OR: 0.69; 95%CI: 0.56–0.84) and girls (OR: 0.82; 95%CI: 0.71–0.94). Migrant condition and geographic area seemed to be other predictors for well-being: being a migrant was associated with higher levels of HC among 11 years old boys. We also observed a northern–southern gradient in the association with well-being: higher levels of LS were found in southern regions, especially in males, in conjunction with more HCs, somatic and psychological (feeling nervous, irritability and feeling low), both in boys and girls.

## 4. Discussion

In the last decades, an unclear pattern in time trend of mental well-being was observed, with high heterogeneity among European countries [16,17]. Although there is some prior evidence of a slight decline in mental well-being in Italy [16], country-specific studies are needed to better understand circumstances, processes, and mechanisms involved in this complex public health issue.

The present study investigated time trends in adolescent mental well-being between 2010 and 2018 in Italy.

Globally, the findings of the present study enforce the Dual Factor Model conceptualization of well-being [50,51] as a multidimensional construct, in which the different components (i.e., psychological HC and life satisfaction) can have different but correlated temporal trends. According to this, good mental health is achieved just when two conditions are both satisfied: the presence of well-being and positive functioning and the absence of mental illness [52].

Our data showed that older adolescents have a higher vulnerability to mental health problems than the youngers, and this difference increases across years, as in Potrebny [20]. As suggested by some authors, a possible hypothesis that can explain this phenomenon lies in the observed decrease in the age of onset of puberty over the last decades. That aspect might expose early maturing adolescents, especially girls [53], to a higher risk of psychopathology, as depression [54].

Moreover [10,11,16,21,24,45,55], girls seem to be at higher risk of psychosomatic health complaints and lower life satisfaction than boys and this gender gap is growing over time [24]. They also seem to be affected by mental health problems earlier and most marked in life. Why it happens is not yet completely understood. Possible explanations, beyond those exposed before, could be related to the tendency of girls to experience more internalizing than externalizing symptoms [56], but also to more restricted gender rules and higher levels of body dissatisfaction among females, and higher perception of stress related to school performance [24,57,58,59].

In support of this evidence, our results confirmed the strong association between schoolwork pressure and well-being indicators in accordance with Högberg [24]. Intending to give a partial explanation, some authors focused on the negative effects of grading systems with higher educational expectations: these effects could be directly associated with school-related stress and academic self-esteem and indirectly to psychosomatic symptoms and life satisfaction [57,58]. That issue partially justifies why performance stress is increasing health complaints in recent years. However, school stress accounts for a portion of increasing Italian trends, as shown in the present study [16,24].

A second major environmental factor involved in well-being is social support [24,27], both from family and peers, which can also be a crucial protective factor during economic downturns [28].

In agreement with a Scottish study among 13- and 15-year olds [60], we found that those from two-parent families had higher life satisfaction and fewer health complaints compared to adolescents from nontraditional families. However, it was observed that both well-being indicators were more strongly associated with parent–child communication than with family structure or family affluence, as also shown in Levin [60].

According to literature, the family setting could influence well-being not just directly influencing individual relationships but also as a safe place to learn to handle the school environment and adversity [61,62]. Moreover, positive peer support also seemed to benefit psychosomatic complaints [63,64]. Among possible explanations, this kind of support may moderate the negative effects of academic stress [64]. However, adolescents with better communication in both social contexts showed minor psychological complaints [65].

The trend of psychosomatic health complaints has overall increased in the last few years [17,24]. While this trend seems to follow a stable global pattern in Italy between 2001 and 2010 [66], at the European level, a weak increase in psychosomatic symptoms was noted during the period 1993–2010 [10]. In agreement with CFA, our assessment focused on different trends of the two factors of health complaints considered separately (i.e., somatic and psychological health complaints).

On the one hand, the trend of somatic health complaints is controversial: while among 11-year olds, there is a slight decreasing movement over time, among 13- and 15-year-old girls, it is weakly escalating. This minute growth is in line with previous evidence at the European level in 1994–2010 among 15-year olds, where a modest increment in health complaints has been observed in both gender groups [10,11,20]. A possible explanation for higher levels of somatic health complaints in recent years could be found in the increasing use of computers and smartphones (e.g., on social media and online chatting), especially during mid adolescence [67,68]. Such screen-based activities have been found to be associated with weekly backache and headache among adolescents [69]. This hypothesis could partially account for the divergent trend between younger and older adolescents.

On the other hand, the present study revealed that psychological health complaints are more prevalent and increasing at a greater rate over time than somatic ones. This significant growth in psychological health complaints in the last decade is similar to other European countries [10,18,19,20,21,55]. Our results showed that the escalating trend in psychological symptoms in the last years is due mainly to feeling nervous and sleeping difficulties in both sexes and irritability, especially in girls. However, feeling low seems to have a stable development over the years. The forces driving this trend are not yet fully understood.

Again, exposure to social media and digital technology has rapidly increased in the past decade, and a growing body of evidence suggests that this binge media consumption may harm mental health [67,70,71,72,73,74] for all the psychological issues included in the present work [67,73]. In particular, for sleeping difficulties, the strength of the association between screen time and sleep-onset difficulties increased over time, which may reflect a change in screen time (e.g., the increased use of easily accessible screens such as smartphones and tablets) [75].

Understanding why in our study irritability, being nervous, and sleeping difficulties are growing more than feeling low will request further research.

Despite growing health complaints, no overall trends in life satisfaction emerged in the examined period. In agreement with some studies performed in other European countries, overall good life satisfaction was observed in the last two decades [22,23]. However, a slight decrease in 2014 compared to 2010 was figured out, maybe reflecting the worldwide economic deflection that struck Italian families hard [28,31].

Beyond the Dual Factor Model formalization, the observation of different trends of LS and psychological HC in the last decade suggests a breaking up of the connection between cognitive and physical well-being. Further research is needed to better understand this deep change in our adolescents’ lives.

### Strengths and Limitations

This study has several strengths, such as the large, nationally representative sample of Italian adolescents, a standardized international protocol for data collection and the focus on recent data.

Limitations include the cross-sectional and self-report nature of the data. The variables used were restricted to those available in the HBSC study and focused on specific elements of mental well-being. Particularly, our study would have benefitted from the inclusion of other mental well-being outcomes, such as anxiety, depression, behavioral or conduct problems, as these elements would have provided us with a complete picture of time trends in adolescent mental health. In addition, studies including a wider time period could provide a more comprehensive picture of the topic. Further research using HBSC data from the 2022 survey that includes a wider range of time, more mental health outcome measures and other potential determinants of adolescent mental health trends, such as social media use, could help to better understand this complex issue.

## 5. Conclusions

Overall, the present study showed an increasing trend of psychosomatic health complaints from 2010 to 2018, mainly for psychological symptoms, whereas life satisfaction was steady.

High school work pressure and poor social support (family and classmates) seem to play a central role in worsening well-being outcomes.

Our findings require further research to investigate this change in the well-being of Italian adolescents and what could be capable of breaking up the connection between psychophysical symptomatology and cognitive perception of life satisfaction.

## Figures and Tables

**Table 1:** Descriptive characteristics (%) of the total sample and across surveys.

	2010 (*n* = 58,928)	2014 (*n* = 47,799)	2018 (*n* = 58,976)	Overall (*n* = 165,703)
Males	50.1	50.5	50.7	50.4
Age				
11 years	35.7	36.4	36.6	36.2
13 years	34.5	35.2	36.0	35.3
15 years	29.8	28.4	27.5	28.5
FAS				
Low	13.3	24.7	22.3	20.0
Medium	43.9	51.7	51.0	48.9
High	42.9	23.6	26.7	31.1
Geographic area				
Northern	34.1	41.4	45.6	40.5
Centre	17.5	16.4	17.6	17.2
Southern	48.4	42.2	36.8	42.3
Immigration background	10.6	14.1	13.2	12.6
High school work pressure	41.8	51.3	54.9	49.4
Good classmate support	81.0	74.9	75.7	77.2
Nontraditional family structure	14.7	15.7	18.0	16.2
Good mother support	81.4	81.2	80.0	80.8
Good father support	61.1	66.1	63.9	63.7
LS	34.4	28.7	30.9	31.4
HC	47.3	47.5	52.2	49.1
PSY-HC	33.2	36.4	41.7	37.3
SOM-HC	19.1	19.3	20.7	19.7
Health complaints (more than once a week)				
Headache	26.1	24.4	24.7	25.1
Stomachache	15.3	14.2	16.0	15.2
Backache	14.0	16.1	17.1	15.7
Dizziness	13.8	15.5	16.4	15.2
Feeling low	31.7	34.1	34.0	33.3
Irritability	27.1	30.2	35.1	30.9
Feeling nervous	34.5	36.1	42.6	37.9
Sleeping difficulty	16.5	19.5	25.3	20.6

Abbreviations. LS: life satisfaction ≥ 9; HC: ≥2 health complaints more than once a week; PSY-HC: ≥2 psychological health complaints more than once a week; SOM-HC: ≥2 somatic health complaints more than once a week; FAS: family affluence scale.

**Table 2:** Trend 2010–2018 of LS, HC, PSY-HC, and SOM-HC, stratified by gender and age groups. Results are reported as OR (95%CI).

	Males			Females		
	11 years	13 years	15 years	11 years	13 years	15 years
**LS**						
Overall trend from 2010 to 2018 (per year)	0.99 (0.98–1.00)	1.00 (0.99–1.01)	0.98 (0.97–1.00)	0.99 (0.97–1.00)	1.00 (0.99–1.02)	1.00 (0.98–1.02)
Survey						
2014 vs. 2010	**0.84** (0.74–0.94)	**0.85** (0.76–0.95)	**0.75** (0.65–0.88)	**0.81** (0.72–0.91)	**0.79** (0.69–0.91)	**0.80** (0.67–0.95)
2018 vs. 2010	0.92 (0.83–1.02)	0.99 (0.89–1.09)	**0.97** (0.77–0.99)	0.89 (0.79–1.00)	1.01 (0.89–1.14)	0.97 (0.83–1.13)
**HC**						
Overall trend from 2010 to 2018 (per year)	0.99 (0.98–1.01)	**1.02** (1.00–1.03)	**1.02** (1.01–1.04)	0.99 (0.98–1.01)	**1.03** (1.02–1.04)	**1.04** (1.02–1.05)
Survey						
2014 vs. 2010	**0.70** (0.63–0.78)	0.90 (0.79–1.01)	1.04 (0.93–1.17)	**0.83** (0.72–0.94)	1.11 (0.99–1.24)	**1.17** (1.02–1.33)
2018 vs. 2010	0.92 (0.83–1.02)	**1.13** (1.01–1.26)	**1.20** (1.09–1.32)	0.93 (0.83–1.05)	**1.26** (1.14–1.38)	**1.33** (1.19–1.49)
**SOM-HC**						
Overall trend from 2010 to 2018 (per year)	0.98 (0.96–1.01)	1.01 (0.99–1.03)	1.01 (0.98–1.04)	**0.98** (0.97–1.00)	**1.02** (1.01–1.04)	**1.02** (1.00–1.04)
Survey						
2014 vs. 2010	**0.67** (0.57–0.78)	0.88 (0.75–1.03)	0.85 (0.66–1.10)	0.93 (0.82–1.05)	**1.17** (1.04–1.31)	1.15 (0.98–1.36)
2018 vs. 2010	**0.86** (0.74–0.99)	1.04 (0.91–1.19)	1.07 (0.85–1.33)	**0.86** (0.76–0.96)	**1.20** (1.09–1.33)	**1.20** (1.03–1.39)
**PSY-HC**						
Overall trend from 2010 to 2018 (per year)	**1.02** (1.00–1.03)	**1.03** (1.02–1.05)	**1.04** (1.02–1.05)	**1.03** (1.01–1.05)	**1.05** (1.04–1.07)	**1.06** (1.05–1.07)
Survey						
2014 vs. 2010	**0.85** (0.76–0.95)	0.94 (0.83–1.07)	1.08 (0.95–1.22)	0.96 (0.83–1.11)	**1.24** (1.10–1.39)	**1.40** (1.25–1.57)
2018 vs. 2010	**1.13** (1.02–1.25)	**1.26** (1.13–1.41)	**1.33** (1.20–1.48)	**1.26** (1.11–1.44)	**1.48** (1.32–1.65)	**1.56** (1.42–1.72)

In bold if *p* ≤ 0.05. Abbreviations. LS: life satisfaction ≥ 9; HC: ≥2 health complaints more than once a week; PSY-HC: ≥2 psychological health complaints more than once a week; SOM-HC: ≥2 somatic health complaints more than once a week. Adjustment variables: survey year, family affluence scale, schoolwork pressure, classmate support, mother support, father support, family structure, immigration background, geographic area

## Data Availability

The data presented in this study are available in accordance with the Italian HBSC data access policy. Requests should be directed to paola.nardone@iss.it, member of the National Centre for Disease Prevention and Health Promotion, Italian National Institute of Health.

## Appendix A

Figure A1. Confirmatory Factor Analysis on health complaints. Results from the double factor (PSY-HC and SOM-HC) model for the dichotomized variable. Standardized coefficients of correlation between latent and observed variables; Table A1. Confirmatory factor analysis (SEM, GSEM). Measures of fit (AIC, BIC) between a single factor (HC) and a double factor (PSY-HC and SOM-HC) model for health complaints, both dichotomized variable and score; Table A2. Prevalence (%) of outcome variables across surveys stratified by gender and age groups; Table A3. Multivariable logistic regression (OR (95%CI)) predicting feeling low, irritability, feeling nervous, and sleeping difficulty (PSY-HC), stratified by gender and age groups; Table A4. Multivariable logistic regression (OR (95%CI)) predicting LS, HC, PSY-HC, and SOM-HC, stratified by gender and age groups.

**Table A1:** Confirmatory factor analysis (SEM, GSEM). Measures of fit (AIC, BIC) between a single factor (HC) and a double factor (PSY-HC and SOM-HC) model for health complaints, both dichotomized variable and score.

	AIC	BIC	df
**Continuous (SEM)**			
single factor	4,101,076	4,101,316	24
double factor	4,057,805	4,058,056	25
**Dichotomized (GSEM)**			
single factor	1,171,920	1,172,160	24
double factor	1,141,340	1,141,591	25

Abbreviations. SEM: Structural equation modeling; GSEM: Generalized Structural Equation Modeling; AIC: Akaike Information Criterion; BIC: Bayesian Information Criterion; df: degrees of freedom; HC: ≥2 health complaints more than once a week; PSY-HC: ≥2 psychological health complaints more than once a week; SOM-HC: ≥2 somatic health complaints more than once a week.

**Table A2:** Prevalence (%) of outcome variables across surveys stratified by gender and age groups.

	Males				Females			
	2010	2014	2018	Overall	2010	2014	2018	Overall
**LS**								
Age								
11 years	47.8	40.9	43.0	44.0	49.5	42.3	44.2	45.3
13 years	34.4	29.5	31.4	31.8	29.6	22.5	25.7	26.0
15 years	24.1	18.8	20.3	21.1	16.5	12.3	13.6	14.2
**HC**								
Age								
11 years	41.7	36	42.9	40.4	48.4	46.4	50.7	48.6
13 years	36.5	35.3	41.5	38.0	55.9	61.3	64.3	60.6
15 years	38.2	39.4	44.2	40.7	63.5	69.9	72.9	68.7
**PSY-HC**								
Age								
11 years	26.7	25.4	31.5	28.0	28.6	30.5	37.6	32.5
13 years	25.6	26.1	32.1	28.2	40.8	49.9	54.1	48.4
15 years	29.6	30.5	35.9	32.1	49.1	60.1	63.3	57.3
**SOM-HC**								
Age								
11 years	16.9	12.3	15.2	14.9	23.6	22.8	22.6	23.0
13 years	12.1	11.2	13.7	12.4	23.1	27.5	28.2	26.3
15 years	11.5	11.2	13.6	12.1	27.3	31.8	32.1	30.3

Abbreviations. LS: life satisfaction ≥ 9; HC: ≥2 health complaints more than once a week; PSY-HC: ≥2 psychological health complaints more than once a week; SOM-HC: ≥2 somatic health complaints more than once a week.

**Table A3:** Multivariable logistic regression (OR (95%CI)) predicting feeling low, irritability, feeling nervous and sleeping difficulty (PSY-HC), stratified by gender and age groups.

	Males			Females		
	11 Years	13 Years	15 Years	11 Years	13 Years	15 Years
**Feeling** **low**						
Overall trend from 2010 to 2018 (per year)	0.99 (0.98–1.01)	1.00 (0.98–1.01)	1.00 (0.98–1.01)	1.00 (0.98–1.02)	1.00 (0.98–1.01)	1.01 (0.99–1.02)
Survey						
2014 vs. 2010	0.93 (0.82–1.06)	0.99 (0.88–1.13)	0.97 (0.85–1.11)	0.98 (0.83–1.16)	1.07 (0.95–1.22)	**1.23** (1.08–1.40)
2018 vs. 2010	0.93 (0.83–1.04)	0.99 (0.88–1.12)	0.98 (0.87–1.10)	1.02 (0.87–1.19)	0.99 (0.88–1.11)	1.06 (0.94–1.18)
**Irritability**						
Overall trend from 2010 to 2018 (per year)	**1.03** (1.01–1.04)	**1.03** (1.02–1.04)	**1.03** (1.02–1.05)	**1.03** (1.02–1.04)	**1.06** (1.04–1.08)	**1.07** (1.05–1.08)
Survey						
2014 vs. 2010	0.89 (0.78–1.01)	0.95 (0.84–1.07)	0.99 (0.87–1.12)	1.09 (0.96–1.23)	**1.30** (1.12–1.51)	**1.32** (1.19–1.48)
2018 vs. 2010	**1.17** (1.05–1.31)	**1.21** (1.08–1.35)	**1.27** (1.14–1.40)	**1.26** (1.13–1.40)	**1.62** (1.41–1.85)	**1.68** (1.53–1.85)
**Feeling** **nervous**						
Overall trend from 2010 to 2018 (per year)	**1.02** (1.00–1.04)	**1.03** (1.01–1.04)	**1.04** (1.02–1.06)	**1.03** (1.01–1.04)	**1.05** (1.04–1.07)	**1.05** (1.04–1.07)
Survey						
2014 vs. 2010	**0.77** (0.67–0.88)	0.81 (0.80–1.03)	1.03 (0.90–1.18)	**0.89** (0.79–0.99)	**1.23** (1.07–1.41)	**1.14** (1.01–1.28)
2018 vs. 2010	**1.14** (1.00–1.30)	**1.21** (1.07–1.36)	**1.34** (1.20–1.51)	**1.22** (1.10–1.34)	**1.51** (1.33–1.72)	**1.46** (1.31–1.61)
**Sleeping** **difficulty**						
Overall trend from 2010 to 2018 (per year)	**1.03** (1.00–1.06)	**1.04** (1.02–1.06)	**1.08** (1.06–1.10)	**1.04** (1.01–1.07)	**1.09** (1.07–1.10)	**1.07** (1.06–1.09)
Survey						
2014 vs. 2010	**0.83** (0.71–0.97)	1.07 (0.92–1.25)	**1.27** (1.07–1.49)	1.01 (0.79–1.28)	**1.37** (1.17–1.60)	**1.32** (1.17–1.48)
2018 vs. 2010	**1.22** (1.05–1.41)	**1.31** (1.14–1.51)	**1.79** (1.56–2.07)	**1.37** (1.10–1.71)	**1.91** (1.65–2.21)	**1.75** (1.58–1.94)

In bold if *p* ≤ 0.05. Abbreviations. PSY-HC: ≥2 psychological health complaints more than once a week. Adjustment variables: survey year, family affluence scale, schoolwork pressure, classmate support, mother support, father support, family structure, immigration background, geographic area.

**Table A4:** Multivariable logistic regression (OR (95%CI)) predicting LS, HC, PSY-HC, and SOM-HC, stratified by gender and age groups.

	Males			Females		
	11 Years	13 Years	15 Years	11 Years	13 Years	15 Years
**LS**						
**Survey**						
2014 vs. 2010	**0.84** (0.74–0.94)	**0.85** (0.76–0.95)	**0.75** (0.65–0.88)	**0.81** (0.72–0.91)	**0.79** (0.69–0.91)	**0.80** (0.67–0.95)
2018 vs. 2010	0.92 (0.83–1.02)	0.99 (0.89–1.09)	**0.97** (0.77–0.99)	0.89 (0.79–1.00)	1.01 (0.89–1.14)	0.97 (0.83–1.13)
**FAS**						
Middle vs. low	**1.15** (1.00–1.33)	**1.27** (1.11–1.45)	**1.42** (1.12–1.79)	1.09 (0.97–1.23)	**1.45** (1.24–1.69)	**1.27** (1.04–1.56)
High vs. low	**1.48** (1.31–1.68)	**1.46** (1.23–1.74)	**1.85** (1.49–2.30)	**1.33** (1.16–1.52)	**1.73** (1.48–2.03)	**1.55** (1.26–1.90)
**High school work pressure**	**0.60** (0.55–0.66)	**0.71** (0.65–0.79)	**0.74** (1.67–0.82)	**0.64** (0.58–0.70)	**0.62** (0.56–0.70)	**0.63** (0.55–0.72)
**Nontraditional family structure**	**0.64** (0.57–0.72)	**0.70** (0.62–0.79)	0.89 (0.7–1.08)	**0.68** (0.58–0.81)	**0.60** (0.49–0.71)	0.84 (0.63–1.11)
**Good mother support**	**1.35** (1.14–1.60)	**1.59** (1.37–1.84)	**1.82** (1.53–2.16)	**1.71** (1.45–2.00)	**2.22** (1.79–2.76)	**2.21** (1.90–2.58)
**Good father support**	**1.58** (1.34–1.86)	**1.86** (1.66–2.08)	**1.83** (1.59–2.10)	**2.09** (1.89–2.30)	**2.08** (1.87–2.30)	**1.97** (1.72–2.25)
**Good classmate support**	**2.06** (1.75–2.44)	**1.89** (1.66–2.14)	**1.82** (1.57–2.11)	**2.11** (1.85–2.40)	**1.82** (1.62–2.06)	**1.65** (1.41–1.93)
**Geographic area**						
Centre vs. northern	**1.15** (1.04–1.28)	**1.17** (1.04–1.32)	1.10 (0.96–1.27)	1.07 (0.94–1.21)	1.07 (0.94–1.22)	0.93 (0.79–1.08)
Southern vs. northern	**1.21** (1.09–1.35)	**1.46** (1.23–1.74)	**1.19** (1.04–1.36)	**1.20** (1.07–1.34)	1.12 (0.99–1.27)	1.09 (0.93–1.26)
**Immigration background**	0.91 (0.79–1.05)	0.95 (0.82–1.09)	1.16 (0.96–1.41)	0.91 (0.80–1.03)	0.89 (0.77–1.03)	1.01 (0.83–1.24)
**HC**						
**Survey**						
2014 vs. 2010	**0.70** (0.63–0.78)	0.90 (0.79–1.01)	1.04 (0.93–1.17)	**0.83** (0.72–0.94)	1.11 (0.99–1.24)	**1.17** (1.02–1.33)
2018 vs. 2010	0.92 (0.83–1.02)	**1.13** (1.01–1.26)	**1.20** (1.09–1.32)	0.93 (0.83–1.05)	**1.26** (1.14–1.38)	**1.33** (1.19–1.49)
**FAS**						
Middle vs. low	**0.81** (0.72–0.91)	0.96 (0.85–1.09)	0.89 (0.77–1.04)	**0.88** (0.77–0.99)	**0.82** (0.71–0.94)	**0.85** (0.74–0.97)
High vs. low	**0.77** (0.67–0.89)	**0.84** (0.73–0.95)	**0.84** (0.71–0.98)	**0.83** (0.72–0.96)	**0.77** (0.67–0.88)	**0.75** (0.64–0.88)
**High school work pressure**	**2.50** (2.29–2.74)	**2.15** (1.95–2.37)	**2.21** (2.03–2.41)	**2.64** (2.40–2.90)	**2.55** (2.32–2.80)	**2.22** (2.00–2.47)
**Nontraditional family structure**	**1.29** (1.12–1.49)	**1.37** (1.19–1.58)	**1.23** (1.06–1.44)	**1.35** (1.19–1.53)	**1.25** (1.09–1.44)	**1.20** (1.02–1.41)
**Good mother support**	**0.77** (0.66–0.90)	**0.77** (0.68–0.87)	**0.67** (0.59–0.76)	**0.69** (0.57–0.79)	**0.56** (0.45–0.70)	**0.57** (0.51–0.64)
**Good father support**	**0.63** (0.56–0.71)	**0.62** (0.54–0.72)	**0.59** (0.54–0.66)	**0.56** (0.51–0.61)	**0.49** (0.44–0.55)	**0.66** (0.59–0.73)
**Good classmate support**	**0.49** (0.43–0.55)	**0.50** (0.45–0.55)	**0.51** (0.46–0.56)	**0.54** (0.46–0.63)	**0.61** (0.56–0.67)	**0.64** (0.57–0.73)
**Geographic area**						
Centre vs. northern	**1.24** (1.11–1.40)	1.10 (0.98–1.24)	1.08 (0.98–1.20)	**1.19** (1.07–1.33)	**1.19** (1.07–1.33)	**1.36** (1.22–1.53)
Southern vs. northern	**1.35** (1.22–1.49)	**1.19** (1.06–1.33)	**1.24** (1.06–1.44)	**1.53** (1.36–1.72)	**1.59** (1.44–1.76)	**1.63** (1.44–1.85)
**Immigration background**	**1.17** (1.02–1.35)	0.90 (0.79–1.04)	1.03 (0.89–1.19)	0.98 (0.85–1.12)	1.10 (0.91–1.33)	1.04 (0.87–1.24)
**SOM-HC**						
**Survey**						
2014 vs. 2010	**0.67** (0.57–0.78)	0.88 (0.75–1.03)	0.85 (0.66–1.10)	0.93 (0.82–1.05)	**1.17** (1.04–1.31)	1.15 (0.98–1.36)
2018 vs. 2010	**0.86** (0.74–0.99)	1.04 (0.91–1.19)	1.07 (0.85–1.33)	**0.86** (0.76–0.96)	**1.20** (1.09–1.33)	**1.20** (1.03–1.39)
**FAS**						
Middle vs. low	**0.79** (0.67–0.93)	0.98 (0.83–1.18)	**0.75** (0.62–0.90)	**0.84** (0.74–0.97)	0.95 (0.92–1.10)	**0.83** (0.72–0.94)
High vs. low	0.85 (0.71–1.02)	0.89 (0.73–1.09)	**0.69** (0.56–0.84)	**0.86** (0.75–1.00)	0.95 (0.83–1.08)	**0.82** (0.71–0.94)
**High school work pressure**	**1.91** (1.65–2.21)	**1.92** (1.63–2.27)	**1.74** (1.53–1.99)	**1.89** (1.69–2.11)	**1.87** (1.68–2.09)	**1.79** (1.61–2.00)
**Nontraditional family structure**	1.12 (0.95–1.33)	**1.39** (1.13–1.71)	1.12 (0.87–1.43)	**1.31** (1.10–1.57)	**1.30** (1.09–1.55)	1.02 (0.85–1.23)
**Good mother support**	**0.79** (0.66–0.96)	**0.83** (0.71–0.98)	0.90 (0.77–1.05)	**0.74** (0.61–0.89)	**0.75** (0.65–0.87)	**0.78** (0.71–0.86)
**Good father support**	**0.84** (0.71–0.99)	**0.80** (0.69–0.92)	**0.73** (0.63–0.86)	**0.74** (0.66–0.83)	**0.69** (0.63–0.77)	**0.77** (0.70–0.85)
**Good classmate support**	**0.74** (0.64–0.87)	**0.66** (0.56–0.77)	**0.63** (0.53–0.73)	**0.79** (0.68–0.92)	**0.72** (0.64–0.81)	**0.78** (0.70–0.86)
**Geographic area**						
Centre vs. northern	**1.21** (1.03–1.43)	1.08 (0.93–1.29)	1.11 (0.95–1.30)	**1.28** (1.13–1.45)	1.10 (0.96–1.25)	**1.30** (1.15–1.46)
Southern vs. northern	**1.63** (1.42–1.88)	**1.30** (1.12–1.51)	**1.22** (1.02–1.47)	**1.65** (1.46–1.87)	**1.53** (1.38–1.69)	**1.41** (1.24–1.59)
**Immigration background**	1.09 (0.90–1.32)	0.92 (0.76–1.11)	1.13 (0.92–1.38)	1.01 (0.86–1.19)	1.03 (0.87–1.21)	1.10 (0.94–1.27)
**PSY-HC**						
**Survey**						
2014 vs. 2010	**0.85** (0.76–0.95)	0.94 (0.83–1.07)	1.08 (0.95–1.22)	0.96 (0.83–1.11)	**1.24** (1.10–1.39)	**1.40** (1.25–1.57)
2018 vs. 2010	**1.13** (1.02–1.25)	**1.26** (1.13–1.41)	**1.33** (1.20–1.48)	**1.26** (1.11–1.44)	**1.48** (1.32–1.65)	**1.56** (1.42–1.72)
**FAS**						
Middle vs. low	**0.87** (0.77–0.99)	1.00 (0.87–1.15)	1.01 (0.87–1.18)	0.91 (0.79–1.04)	**0.86** (0.77–0.97)	0.99 (0.86–1.13)
High vs. low	**0.85** (0.73–0.98)	**0.84** (0.72–0.97)	1.02 (0.86–1.20)	**0.87** (0.75–1.00)	**0.85** (0.74–0.97)	**0.85** (0.74–0.99)
**High school work pressure**	**2.54** (2.30–2.81)	**2.15** (1.95–2.38)	**2.20** (2.01–2.42)	**2.67** (2.60–2.97)	**2.49** (2.26–2.74)	**2.29** (2.04–2.57)
**Nontraditional family structure**	**1.25** (1.08–1.44)	**1.37** (1.18–1.59)	**1.21** (1.05–1.40)	**1.28** (1.11–1.48)	**1.23** (1.06–1.43)	**1.30** (1.11–1.51)
**Good mother support**	**0.79** (0.68–0.92)	**0.73** (0.63–0.84)	**0.59** (0.52–0.67)	**0.60** (0.52–0.70)	**0.51** (0.43–0.61)	**0.55** (0.59–0.63)
**Good father support**	**0.59** (0.51–0.67)	**0.62** (0.53–0.72)	**0.58** (0.51–0.65)	**0.53** (0.48–0.58)	**0.53** (0.48–0.59)	**0.65** (0.58–0.73)
**Good classmate support**	**0.47** (0.41–0.54)	**0.45** (0.40–0.51)	**0.48** (0.43–0.53)	**0.51** (0.45–0.58)	**0.57** (0.51–0.63)	**0.65** (0.59–0.72)
**Geographic area**						
Centre vs. northern	**1.20** (1.06–1.34)	1.10 (0.96–1.24))	1.10 (0.99–1.23)	1.06 (0.94–1.19)	1.09 (0.96–1.24)	**1.28** (1.15–1.42)
Southern vs. northern	**1.20** (1.08–1.34)	1.10 (0.98–1.24)	**1.19** (1.06–1.33)	**1.23** (1.08–1.40)	**1.40** (1.26–1.57)	**1.61** (1.44–1.79)
**Immigration background**	**1.18** (1.03–1.36)	0.94 (0.81--1.08)	0.98 (0.84–1.14)	0.98 (0.86–1.13)	1.10 (0.93–1.30)	1.08 (0.92–1.27)

In bold if *p* ≤ 0.05. Abbreviations. LS: life satisfaction ≥ 9; HC: ≥2 health complaints more than once a week; PSY-HC: ≥2 psychological health complaints more than once a week; SOM-HC: ≥2 somatic health complaints more than once a week; FAS: family affluence scale. Adjustment variables: survey year, family affluence scale, schoolwork pressure, classmate support, mother support, father support, family structure, immigration background, geographic area.
